# Supplementary material for: Seawater salt-trapped Pseudomonas aeruginosa survives for years and gets primed for salinity tolerance
Source: BMC Microbiol. 2019 Jun 24;19:142. doi: 10.1186/s12866-019-1499-2 (PMC6591848; doi:10.1186/s12866-019-1499-2)
Supplement: Supplementary file 1 — Table S1. The detected mutations in P. aeruginosa clones ATCC27853 after Whole Genome Sequencing. (PDF 75 kb) [file 12866_2019_1499_MOESM1_ESM.pdf]

Table S1. The detected mutations in *P. aeruginosa* clones ATCC27853 after Whole Genome Sequencing.

**Reference Genome**	**Position**	**Reference nucleotide**	**Clone 1**	**Clone 2**	**Clone 3**	**Clone 4**	**Clone 5**	**WT**	**Locus tag**	**Gene product**	**Effect**
CP015117	1557965	G	A	–	–	–	–	–	A4W92_07285	aromatic amino acid transporter	missense_variant c.400C>T p.Leu134Phe
CP015117	2248912	T	–	–	G	–	–	G	A4W92_10450	prepilin-type N-terminal cleavage/methylation domain-containing protein	missense_variant c.211_212delTAinsGC p.Tyr71Ala
CP015117	2248913	A	–	–	C	–	–	C	A4W92_10450	prepilin-type N-terminal cleavage/methylation domain-containing protein	missense_variant c.211_212delTAinsGC p.Tyr71Ala
CP015117	3593639	G	C	C	C	–	–	C	A4W92_16615	DNA polymerase III subunit beta	synonymous_variant c.795G>C p.Arg265Arg
CP015117	3695540	G	–	C	–	–	–	–	A4W92_17055	FHA domain-containing protein	missense_variant c.338C>G p.Ala113Gly
CP015117	4059537	C	T	T	–	–	–	T	A4W92_18780	hybrid sensor histidine kinase/response regulator	missense_variant c.4204C>T p.Arg1402Cys
CP015117	4461847	A	A	G				G	intergenic		
CP015117	5180188	A	A	T	A	A		A	intergenic		
CP015117	5282094	C	–	–	–	–	T	T	intergenic		
CP015117	6810389	T	–	C	C	–	–	C	A4W92_31605	hypothetical protein	missense_variant c.16T>C p.Ser6Pro
CP015117	6810407	T	–	C	C	–	–	C	A4W92_31605	hypothetical protein	synonymous_variant c.34T>C p.Leu12Leu
CP015117	6810427	C	–	–	T	–	–	T	A4W92_31605	hypothetical protein	synonymous_variant c.54C>T p.Ser18Ser
CP015117	6810434	T	–	T	G	–	–	G	A4W92_31605	hypothetical protein	missense_variant c.61T>G p.Cys21Gly

-Clone 1 to Clone 5: *P. aeruginosa* Biosamples available from NCBI database under accession numbers: SAMN08127309; SAMN08127310; SAMN08127311; SAMN08127312 and SAMN08127313.

-WT: wild-type strain
