# Supplementary figures and images for: Seawater salt-trapped Pseudomonas aeruginosa survives for years and gets primed for salinity tolerance
Source: BMC Microbiol. 2019 Jun 24;19:142. doi: 10.1186/s12866-019-1499-2 (PMC6591848; doi:10.1186/s12866-019-1499-2)

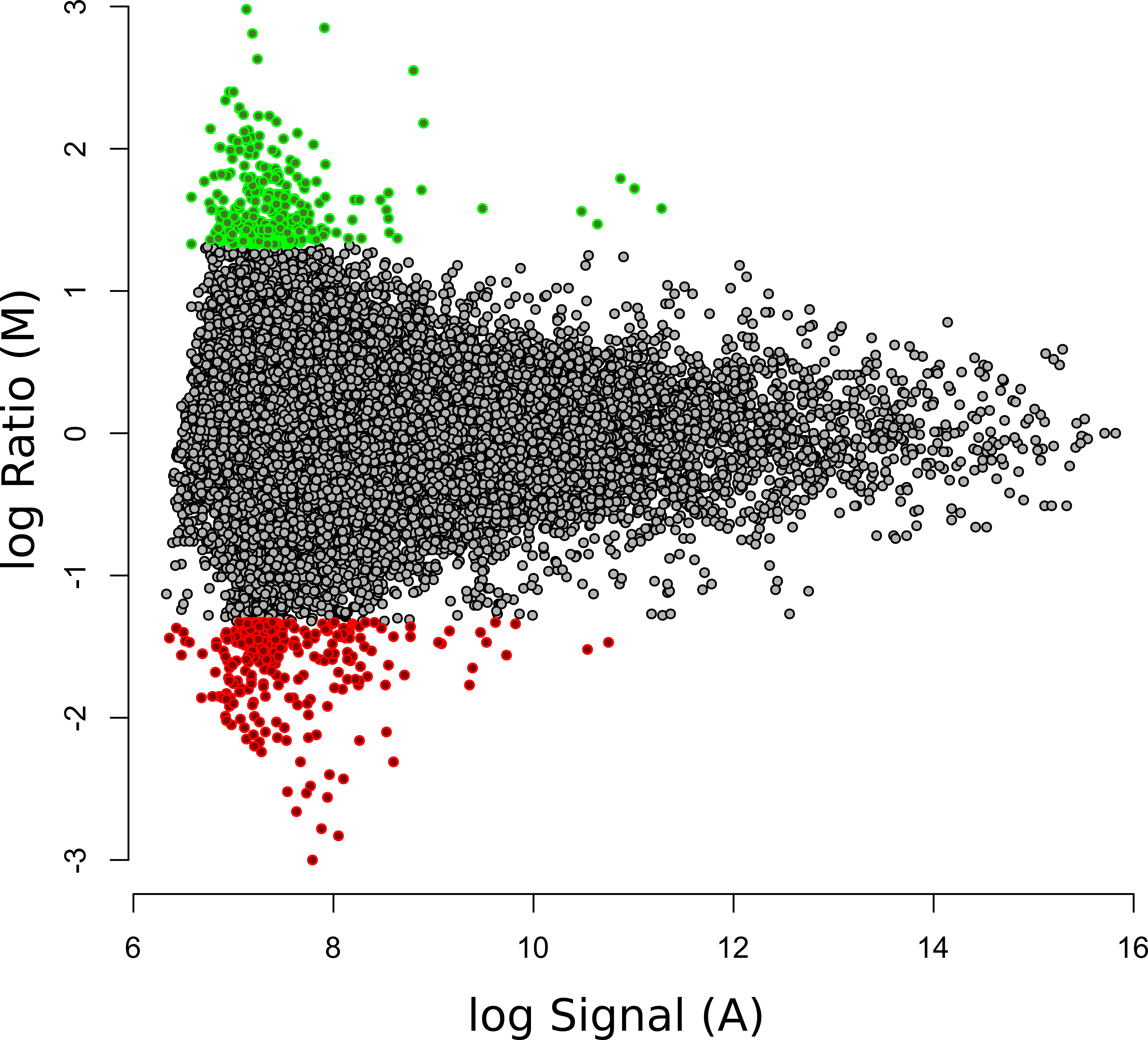

Supplement: Supplementary file 2 — Figure S1. (PNG 1930 kb) [file 12866_2019_1499_MOESM2_ESM.png]
