# Supplementary material for: Seawater salt-trapped Pseudomonas aeruginosa survives for years and gets primed for salinity tolerance
Source: BMC Microbiol. 2019 Jun 24;19:142. doi: 10.1186/s12866-019-1499-2 (PMC6591848; doi:10.1186/s12866-019-1499-2)
Supplement: Supplementary file 3 — Table S2. Gene list of mutants recovered from the P. aeruginosa PA14 mutant insertion library and used in this study to test the impact of salt in the growth. (PDF 74 kb) [file 12866_2019_1499_MOESM3_ESM.pdf]

Table S2. Gene list of mutants recovered from the *P. aeruginosa* PA14 mutant insertion library and used in this study to test the impact of salt in the growth.

**Gene (Pseudomonas ID)**	**Description**
PA0310	Hypothetical protein
PA0547	Probable transcriptional regulator
speD (PA0654)	S-adenosylmethionine decarboxylase proenzyme
rsmA (PA0905)	Ribosomal RNA small subunit methyltransferase A
yehS (PA0915)	Conserved hypothetical protein
shaD (PA1057)	Na^+/H^+ antiporter subunit D
PA1209	Hypothetical protein
fixG (PA1551)	Probable ferredoxin
ccoP2 (PA1555)	cbb3-type cytochrome *c* oxidase, CcoP subunit
ccoO2 (PA1556)	cbb3-type cytochrome *c* oxidase, CcoO subunit
ccoN2 (PA1557)	cbb3-type cytochrome *c* oxidase, CcoN subunit
pcr3 (PA1701)	YscX family type III secretion protein, preprotein translocase X
pcrV (PA1706)	Type III secretion protein PcrV
pcrH (PA1707)	Regulatory protein PcrH
popB (PA1708)	Translocator protein PopB
popD (PA1709)	Translocator outer membrane protein PopD precursor
exsB (PA1712)	Exoenzyme S synthesis protein B
PA2501	Hypothetical protein
PA2662	Conserved hypothetical protein
PA2757	Hypothetical protein
uup (PA3019)	Probable ATP-binding component of ABC transporter
gapA (PA3195)	Glyceraldehyde 3-phosphate dehydrogenase
bfrB (PA3531)	Bacterioferritin
trmD (PA3743)	tRNA (guanine-N1)-methyltransferase
iscS (PA3814)	Pyridoxal phosphate-dependent L-cysteine desulfurase
iscR (PA3815)	Iron sulfur biosynthesis cluster operon transcriptional regulator IscR
ispA (PA4043)	Geranyltranstransferase
oprG (PA4067)	Outer membrane protein OprG precursor
PA4390	Hypothetical protein
cysD (PA4443)	ATP sulfurylase small subunit
PA4517	Conserved hypothetical protein
PA4611	Hypothetical protein

yjjT (PA4627)	Conserved hypothetical protein
ftsJ (PA4752)	Cell division protein FtsJ
dnaK (PA4761)	Chaperone protein DnaK
prmA (PA4850)	Ribosomal protein L11 methyltransferase
hslU (PA5054)	Heat shock protein HslU
PA5174	Probable beta-ketoacyl synthase
PA5530	C5-dicarboxylate transporter
