# Supplementary material for: Seawater salt-trapped Pseudomonas aeruginosa survives for years and gets primed for salinity tolerance
Source: BMC Microbiol. 2019 Jun 24;19:142. doi: 10.1186/s12866-019-1499-2 (PMC6591848; doi:10.1186/s12866-019-1499-2)
Supplement: Supplementary file 4 — Table S3. (PDF 725 kb) [file 12866_2019_1499_MOESM4_ESM.pdf]

Gene ID	Gene Name<br>Gene Symbol	PANTHER Family /Subfamily	PANTHER Protein Class
**Up-regulated Genes**			
PA3641|UniProtKB=Q9HXZ0	Probable amino acid permease<br>PA3641	SUBFAMILY NOT NAMED (PTHR30330:SF1)	-
PA0281|UniProtKB=Q9I6K9	Sulfate transport protein CysW<br>*cysW*	Sulfate transport system permease protein CysW (PTHR30406:SF1)	-
PA4850|UniProtKB=Q9HUW3	Ribosomal protein L11 methyltransferase<br>*prmA*	Electron transfer flavoprotein beta subunit lysine methyltransferase (PTHR43648:SF1)	DNA methyltransferase<br>RNA methyltransferase
PA5174|UniProtKB=Q9HU15	Beta-ketoacyl-[acyl-carrier-protein] synthase FabY<br>*fabY*	3-oxoacyl-[acyl-carrier-protein] synthase 1 (PTHR11712:SF306)	acyltransferase<br>dehydrogenase<br>esterase<br>ligase<br>methyltransferase
PA1708|UniProtKB=Q9I324	Translocator protein PopB<br>*popB*	-	-
PA1709|UniProtKB=Q9I323	Translocator outer membrane protein PopD<br>*popD*	-	-
PA1707|UniProtKB=Q9I325	Regulatory protein PcrH<br>*pcrH*	-	-
PA1710|UniProtKB=P26995	Exoenzyme S synthesis protein C<br>*exsC*	-	-
PA0547|UniProtKB=Q9I5Y9	Probable transcriptional regulator<br>PA0547	-	-
PA1228|UniProtKB=Q9I4B0	Uncharacterized protein<br>PA1228	-	-
PA0282|UniProtKB=Q9I6K8	Sulfate transport protein CysT<br>*cysT*	Sulfate transport system permease protein CysT (PTHR30406:SF8)	-
PA1714|UniProtKB=Q9I321	ExsD<br>*exsD*	-	-
PA1701|UniProtKB=G3XD37	Uncharacterized protein<br>PA1701	-	-
PA2749|UniProtKB=Q9I094	DNA-specific endonuclease I<br>*endA*	Endonuclease-1 (PTHR33607:SF2)	-
PA1719|UniProtKB=P95434	Type III export protein PscF<br>*pscF*	-	-
PA4412|UniProtKB=Q9HW01	UDP-N-acetylglucosamine--N-acetylmuramyl-(pentapeptide) pyrophosphoryl-undecaprenol N-acetylglucosamine transferase<br>*murG*	Glycosyltransferase (PTHR21015:SF22)	acetyltransferase<br>glycosyltransferase<br>transfer/carrier protein
PA3814|UniProtKB=Q9HXI8	Cysteine desulfurase IscS<br>*iscS*	Selenocysteine lyase (PTHR11601:SF52)	lyase
PA1695|UniProtKB=	Translocation protein in type III	-	-

Q9I332	secretion<br>*pscP*		
PA2252|UniProtKB=<br>Q9I1L7	Probable AGCS sodium/alanine/glycine symporter<br>PA2252	SUBFAMILY NOT NAMED<br>(PTHR30330:SF1)	-
PA4043|UniProtKB=<br>Q9HWY4	Geranyltranstransferase<br>*ispA*	Geranylgeranyl pyrophosphate synthase 10, mitochondrial-related<br>(PTHR43281:SF1)	acyltransferase
PA5429|UniProtKB=<br>Q9HTD7	Aspartate ammonia-lyase<br>*aspA*	Aspartate ammonia-lyase<br>(PTHR42696:SF2)	lyase
PA1057|UniProtKB=<br>Q9I4R7	Uncharacterized protein<br>PA1057	SUBFAMILY NOT NAMED<br>(PTHR34584:SF1)	-
PA1319|UniProtKB=<br>Q9I425	Cytochrome bo(3) ubiquinol oxidase subunit 3<br>*cyoC*	Cytochrome bo(3) ubiquinol oxidase subunit 3<br>(PTHR11403:SF2)	oxidase
PA3182|UniProtKB=<br>Q9X2N2	6-phosphogluconolactonase<br>*pgl*	6-phosphogluconolactonase<br>(PTHR11054:SF0)	hydrolase
PA3815|UniProtKB=<br>Q9HXI7	IscR<br>*iscR*	HTH-type transcriptional regulator IscR<br>(PTHR33221:SF10)	-
PA3841|UniProtKB=<br>G3XDA1	Exoenzyme S<br>*exoS*	SUBFAMILY NOT NAMED<br>(PTHR10339:SF30)	-
PA2757|UniProtKB=<br>Q9I086	Uncharacterized protein<br>PA2757	-	-
PA3019|UniProtKB=<br>Q9HZI7	Probable ATP-binding component of ABC transporter<br>PA3019	ABC transporter ATP-binding protein uup<br>(PTHR19211:SF69)	-
PA2991|UniProtKB=<br>P57112	Soluble pyridine nucleotide transhydrogenase<br>*sthA*	Soluble pyridine nucleotide transhydrogenase<br>(PTHR22912:SF93)	dehydrogenase<br>oxidase<br>reductase
PA1838|UniProtKB=<br>Q9I2Q7	Sulfite reductase<br>*cysI*	Sulfite reductase [NADPH] subunit beta<br>(PTHR11493:SF47)	-
PA3809|UniProtKB=<br>Q51383	2Fe-2S ferredoxin<br>*fdx*	2Fe-2S ferredoxin<br>(PTHR23426:SF34)	-
PA3131|UniProtKB=<br>Q9HZ93	Probable aldolase<br>PA3131	KHG/KDPG aldolase<br>(PTHR30246:SF0)	aldolase
PA2662|UniProtKB=<br>Q9I0H6	Uncharacterized protein<br>PA2662	-	-
PA1699|UniProtKB=<br>G3XCT8	Uncharacterized protein<br>PA1699	-	-
PA1700|UniProtKB=<br>Q9I329	Uncharacterized protein<br>PA1700	-	-
PA2998|UniProtKB=<br>Q9HZK7	Na(+)-translocating NADH-quinone reductase subunit B<br>*nqrB*	SUBFAMILY NOT NAMED<br>(PTHR30578:SF1)	-
PA0044|UniProtKB=<br>Q9I788	Exoenzyme T<br>*exoT*	SUBFAMILY NOT NAMED	-

		(PTHR10339:SF30)	
PA4442|UniProtKB=O50274	Bifunctional enzyme CysN/CysC<br>*cysNC*	Elongation factor 1-alpha 1-related<br>(PTHR23115:SF170)	G-protein<br>hydrolase<br>translation elongation factor<br>translation initiation factor
PA5012|UniProtKB=G3XD35	Heptosyltransferase II<br>*waaF*	ADP-heptose--LPS heptosyltransferase 2<br>(PTHR30160:SF7)	carbohydrate kinase<br>glycosyltransferase
PA2204|UniProtKB=Q9I1R3	Probable binding protein component of ABC transporter<br>PA2204	-	-
PA1318|UniProtKB=Q9I426	Cytochrome bo(3) ubiquinol oxidase subunit 1<br>*cyoB*	Cytochrome bo(3) ubiquinol oxidase subunit 1<br>(PTHR10422:SF35)	oxidase
PA3811|UniProtKB=Q9HXJ1	Co-chaperone protein HscB homolog<br>*hscB*	Co-chaperone protein HscB<br>(PTHR14021:SF16)	-
PA4002|UniProtKB=G3XD88	Rod shape-determining protein<br>*rodA*	Rod shape-determining protein RodA<br>(PTHR30474:SF1)	-
PA0915|UniProtKB=Q9I542	Uncharacterized protein<br>PA0915	SUBFAMILY NOT NAMED<br>(PTHR37805:SF1)	-
PA0284|UniProtKB=Q9I6K6	Uncharacterized protein<br>PA0284	-	-
PA1706|UniProtKB=G3XD49	Type III secretion protein PcrV<br>*pcrV*	-	-
PA1712|UniProtKB=P26994	Exoenzyme S synthesis protein B<br>*exsB*	-	-
PA4627|UniProtKB=Q9HVG4	Ribosomal RNA small subunit methyltransferase C<br>*rsmC*	Ribosomal RNA small subunit methyltransferase C<br>(PTHR18895:SF70)	DNA methyltransferase<br>RNA methyltransferase
PA0789|UniProtKB=Q9I5E9	Probable amino acid permease<br>PA0789	SUBFAMILY NOT NAMED<br>(PTHR43341:SF2)	-
PA1717|UniProtKB=Q9I318	Type III export protein PscD<br>*pscD*	-	-
PA5530|UniProtKB=Q9HT43	Probable MFS dicarboxylate transporter<br>PA5530	SUBFAMILY NOT NAMED<br>(PTHR43528:SF5)	-
PA1696|UniProtKB=Q9I331	Translocation protein in type III secretion<br>*pscO*	-	-
PA3743|UniProtKB=Q9HXQ1	tRNA (guanine-N(1)-)-methyltransferase<br>*trmD*	tRNA (guanine-N(1)-)-methyltransferase<br>(PTHR32125:SF1)	-
PA1723|UniProtKB=Q9I314	Type III export protein PscJ<br>*pscJ*	SUBFAMILY NOT NAMED<br>(PTHR30046:SF2)	-

PA3195|UniProtKB=P27726	Glyceraldehyde-3-phosphate dehydrogenase<br>*gap*	glyceraldehyde-3-phosphate dehydrogenase C-related (PTHR43148:SF2)	dehydrogenase
PA4390|UniProtKB=Q9HW14	Uncharacterized protein<br>PA4390	-	-
PA1697|UniProtKB=Q9I330	ATP synthase in type III secretion system<br>PA1697	SUBFAMILY NOT NAMED (PTHR15184:SF48)	ATP synthase<br>DNA binding protein<br>anion channel<br>hydrolase<br>ligand-gated ion channel
PA0654|UniProtKB=Q9I5R7	S-adenosylmethionine decarboxylase proenzyme<br>*speD*	S-adenosylmethionine decarboxylase proenzyme (PTHR33866:SF1)	-
PA4443|UniProtKB=O50273	Sulfate adenylyltransferase subunit 2<br>*cysD*	Sulfate adenylyltransferase subunit 2 (PTHR43196:SF1)	nucleotidyltransferase
PA3068|UniProtKB=Q9HZE0	NAD-specific glutamate dehydrogenase<br>*gdhB*	SUBFAMILY NOT NAMED (PTHR43403:SF1)	dehydrogenase
PA0570|UniProtKB=Q9I5W6	Uncharacterized protein<br>PA0570	-	-
**Down-regulated genes**			
PA1557|UniProtKB=Q9I3G0	Cytochrome c oxidase, cbb3-type, CcoN subunit<br>*ccoN2*	SUBFAMILY NOT NAMED (PTHR10422:SF29)	oxidase
PA1555|UniProtKB=Q9I3G2	Cbb3-type cytochrome c oxidase subunit<br>*ccoP2*	SUBFAMILY NOT NAMED (PTHR33751:SF1)	-
PA3531|UniProtKB=Q9HY79	Ferroxidase<br>*bfrB*	Bacterioferritin (PTHR30295:SF0)	storage protein
PA5446|UniProtKB=Q9HTC1	Uncharacterized protein<br>PA5446	-	-
PA4752|UniProtKB=P95454	Ribosomal RNA large subunit methyltransferase E<br>*rlmE*	rRNA methyltransferase 2, mitochondrial (PTHR10920:SF18)	-
PA0310|UniProtKB=Q9I6I1	Uncharacterized protein<br>PA0310	SUBFAMILY NOT NAMED (PTHR12907:SF22)	-
PA3572|UniProtKB=Q9HY48	Uncharacterized protein<br>PA3572	-	-
PA4611|UniProtKB=Q9HVH9	Uncharacterized protein<br>PA4611	-	-
PA0905|UniProtKB=O69078	Carbon storage regulator homolog<br>*csrA*	Carbon storage regulator (PTHR34984:SF1)	-
PA4067|UniProtKB=Q9HWW1	Outer membrane protein OprG<br>*oprG*	Outer membrane protein W (PTHR36920:SF1)	-
PA1556|UniProtKB=Q9I3G1	Cytochrome c oxidase, cbb3-type, CcoO subunit<br>*ccoO2*<br>ortholog	-	-
PA5054|UniProtKB=	ATP-dependent protease ATPase	ATP-dependent protease	chaperone

Q9HUC5	subunit HslU<br>hslU<br>ortholog	ATPase subunit HslU<br>(PTHR43815:SF1)	
PA1551|UniProtKB=<br>Q9I3G6	Probable ferredoxin<br>PA1551<br>ortholog	SUBFAMILY NOT<br>NAMED<br>(PTHR24960:SF45)	-
PA2501|UniProtKB=<br>Q9I0Y1	Uncharacterized protein<br>PA2501<br>ortholog	-	-
PA1209|UniProtKB=<br>Q9I4C9	Uncharacterized protein<br>PA1209<br>ortholog	SUBFAMILY NOT<br>NAMED<br>(PTHR42709:SF7)	-
PA4761|UniProtKB=<br>Q9HV43	Chaperone protein DnaK<br>dnaK<br>ortholog	Stress-70 protein,<br>mitochondrial<br>(PTHR19375:SF184)	-
PA4517|UniProtKB=<br>Q9HVQ5	Uncharacterized protein<br>PA4517<br>ortholog	Phosphoethanolamine<br>transferase EptC<br>(PTHR30443:SF2)	-
